# Liposomal Encapsulation Increases the Efficacy of Azithromycin against *Chlamydia trachomatis*

**DOI:** 10.3390/pharmaceutics14010036

**Published:** 2021-12-24

**Authors:** Anita Bogdanov, László Janovák, Jasmina Vraneš, Tomislav Meštrović, Sunčanica Ljubin-Sternak, Zsuzsanna Cseh, Valéria Endrész, Katalin Burián, Željka Vanić, Dezső P. Virok

**Affiliations:** 1 Department of Medical Microbiology, Albert Szent-Györgyi Health Center and Albert Szent-Györgyi Medical School, University of Szeged, Semmelweis Str. 6, 6725 Szeged, Hungary; varga-bogdanov.anita@med.u-szeged.hu (A.B.); cseh.zsuzs@gmail.com (Z.C.); endresz.valeria@med.u-szeged.hu (V.E.); burian.katalin@med.u-szeged.hu (K.B.); 2 Interdisciplinary Excellence Centre, Department of Physical Chemistry and Materials Science, University of Szeged, Rerrich Béla Sqr. 1, 6720 Szeged, Hungary; laszlo.janovak@gmail.com; 3 Molecular Microbiology Department, Dr. Andrija Štampar Teaching Institute of Public Health, Mirogojska cesta 16, 10000 Zagreb, Croatia; jasmina.vranes@stampar.hr (J.V.); sljsternak@stampar.hr (S.L.-S.); 4 Medical Microbiology Department, University of Zagreb School of Medicine, Šalata 3, 10000 Zagreb, Croatia; 5 Clinical Microbiology and Parasitology Unit, Dr. Zora Profozić Polyclinic, Bosutska 19, 10000 Zagreb, Croatia; tomislav.mestrovic@gmail.com; 6 University Centre Varaždin, University North, Ul. 104. Brigade 3, 42000 Varaždin, Croatia; 7 Department of Pharmaceutical Technology, Faculty of Pharmacy and Biochemistry, University of Zagreb, A. Kovačića 1, 10000 Zagreb, Croatia; zvanic@pharma.hr

**Keywords:** *Chlamydia*, *Chlamydia trachomatis*, elastic liposomes, azithromycin, Tween 80, surface charge, PCR, qPCR, MIC, MBC

## Abstract

*Chlamydia trachomatis* (*C. trachomatis*) is an obligate intracellular bacterium linked to ocular and urogenital infections with potentially serious sequelae, including blindness and infertility. First-line antibiotics, such as azithromycin (AZT) and doxycycline, are effective, but treatment failures have also been reported. Encapsulation of antibiotics in liposomes is considered an effective approach for improving their local effects, bioavailability, biocompatibility and antimicrobial activity. To test whether liposomes could enhance the antichlamydial action of AZT, we encapsulated AZT in different surface-charged elastic liposomes (neutral, cationic and anionic elastic liposomes) and assessed their antibacterial potential against the *C. trachomatis* serovar D laboratory strain as well as the clinical isolate *C. trachomatis* serovar F. A direct quantitative polymerase chain reaction (qPCR) method was used to measure chlamydial genome content 48 h post infection and to determine the recoverable chlamydial growth. All the liposomes efficiently delivered AZT to HeLa 229 cells infected with the laboratory *Chlamydia* strain, exhibiting the minimal inhibitory concentrations (MIC) and the minimal bactericidal concentrations (MBC) of AZT even 4–8-fold lower than those achieved with the free AZT. The tested AZT-liposomes were also effective against the clinical *Chlamydia* strain by decreasing MIC values by 2-fold relative to the free AZT. Interestingly, the neutral AZT-liposomes had no effect on the MBC against the clinical strain, while cationic and anionic AZT-liposomes decreased the MBC 2-fold, hence proving the potential of the surface-charged elastic liposomes to improve the effectiveness of AZT against *C. trachomatis*.

## 1. Introduction

*C. trachomatis* ocular and urogenital serovars are responsible for the acute and chronic inflammations of ocular and urogenital mucosal surfaces. The chronic inflammation could lead to serious complications, such as blindness and infertility. According to a World Health Organization report, 44 countries are affected by trachoma, 137 million people are at risk of the infection and 1.9 million people suffer from infection-related impaired vision or blindness [1]. *C. trachomatis*-caused sexually transmitted infections are estimated to be acquired by 131 million people annually, where mostly young (16–24 years-old) people are affected. For instance, 1,758,668 and 406,406 cases of chlamydia infection were reported in 2018 in the USA and in 26 European countries, respectively [2].

The macrolide antibiotic AZT inhibits protein synthesis by irreversibly binding to the 50S subunit of the bacterial ribosome. AZT is able to accumulate in the host cells reaching a high intracellular concentration [3]; therefore, it can be used to inhibit the obligate intracellular *Chlamydia* species. *C. trachomatis* infections can be treated effectively with a single dose of AZT or a one-week course of another bacterial protein synthesis inhibitor doxycycline. Due to the lesser need of compliance, a single-dose AZT regimen may be preferable over to doxycycline. While both antibiotic regimens are effective, treatment failures have also been reported. Systemic reviews and meta-analyses of clinical trials assessed the AZT effectivity of 96–97%, but the relative risk of treatment failure was 2.45 compared to that for doxycycline [4]. Multiple factors could lead to *C. trachomatis* treatment failures including a higher bacterial load [5], homotypic resistance due to chromosomal mutations, such as the mutations of the 23S rRNA [6], and heterotypic resistance, where a certain sub-population of a *Chlamydia* pool displays higher antimicrobial resistance, but this phenotypic feature is not encoded genetically [7].

Liposomal encapsulation is a promising tool for improving antibiotic efficacy and overcoming microbial resistance, thus reducing treatment failures [8]. Liposomal antibiotics have been proven to enable prolonged and/or controlled release of the encapsulated drug permitting a higher local drug concentrations and a greater efficacy against extracellular and certain facultative intracellular bacteria, such as *Listeria monocytogenes*, *Brucella melitensis*, *Francisella tularensis, Salmonella enterica* serovar Typhimurium, *Mycobacterium avium* complex and *Mycobacterium tuberculosis* [9]. Liposomes are additionally attractive as antimicrobial carriers due to their targeting ability and physiological acceptability [10]. Appropriate adjustment of their physicochemical properties (size, bilayer elasticity/rigidity and surface modification) enables enhanced delivery of the entrapped drug to the targeted/infected cells [11] or bacteria, either by facilitating fusion with bacteria or allowing release of the drug in the close vicinity of the microorganisms [12,13,14]. Improved intracellular delivery into the targeted cells can be achieved by several different mechanisms including passive diffusion of the released drug across the cell membrane, adsorption of liposomes on the cell surface, followed by membrane lipid exchange and release of the drug. Specially designed membrane active liposomes containing fusogenic lipids are proposed to fuse with the cell membrane delivering its cargo into the cytoplasm, while receptor-mediated endocytosis is characteristic for ligand-targeted liposomes [11].

Among the different types of liposomes, elastic (deformable) liposomes have already been proven to improve delivery of entrapped drugs deeper in the skin [15,16] and vaginal tissue [17]. Compared to conventional liposomes, consisting of phospholipids with or without addition of cholesterol, elastic liposomes contain phospholipids and certain lower portions of the edge activator (single chain surfactant) and/or solvent (ethanol, propylene glycol, glycerol), making their bilayers fluid and more squeezable [16,18]. Encapsulation of AZT in anionic elastic liposomes have demonstrated stronger activity against biofilm-forming methicillin-resistant *Staphylococcus aureus* strains (MRSA) than conventional liposomes of the same surface charge [18]. We have previously found that elastic liposomes were also more effective in delivering AZT to the *C. trachomatis* infected HeLa cells than conventional liposomes with rigid bilayers, although their activity was lower than the free drug. It was speculated that such results were due to the faster AZT release from elastic liposomes compared to slower release profile achieved by conventional liposomes and the fact that the released lipophilic drug is more readily taken up via the HeLa cells than liposomes. This was also supported by ex vivo permeation assessment demonstrating increased penetration of the free AZT through the vaginal tissue [19].

Continuing previous research [19], and to improve anti-chlamydial activity of liposomal AZT for local therapy, we have prepared and tested several novel preparations of elastic AZT-liposomes, differing in the surface charge (cationic, neutral and anionic), to select the ones with improved antichlamydial action. Our hypothesis was that by facilitating interaction of the liposomes with the *C. trachomatis* infected HeLa cells increased intracellular AZT delivery could be obtained. Namely, cationic liposomes have been demonstrated to contribute interaction with the negatively surface charged cell membranes [20] as well as bacteria [13,14], and significantly increase the activity of encapsulated drugs against planktonic [21] and biofilm-forming bacteria [10].

## 2. Materials and Methods

### 2.1. Materials

Lipoid E PC S (from egg; phosphatidylcholine content ≥ 96%) (EPC) and egg phosphatidylglycerol (EPG) were generous gifts from Lipoid GmbH (Ludwigshafen, Germany), while AZT in the form of a dihydrate was kindly donated by PLIVA Croatia Ltd. (Zagreb, Croatia). Dimethyldioctadecylammonium bromide (DODAB) and polyoxyethylene(20) sorbitanmonooleate (Tween 80) were provided by Sigma–Aldrich (St. Louis, MO, USA). Ethanol and KH_2_PO_4_ were purchased from Kemika (Zagreb, Croatia). For the preparation of liposomes, 0.01 M phosphate buffer (PB) was prepared by dissolving 1.3609 g KH_2_PO_4_ in demineralized water up to 1000 mL, and the pH was adjusted to 7.5 by the addition of 10 M KOH [19].

### 2.2. C. trachomatis Strains

The laboratory reference strain *C. trachomatis* serovar D (UW-3/CX, ATCC) and a *C. trachomatis* serovar F clinical isolate were used in this study. *C. trachomatis* clinical isolate was obtained from a male urethra sample during routine testing at the Referral Center for Diagnostics of Sexually Transmitted Infections in the Dr. Andrija Štampar Teaching Institute for Public Health, Zagreb, Croatia. The specimen was collected using the collection kit comprised of a Dacron swab and MicroTest M4RT transport medium (Remel Inc., Lenexa, KS, USA) and stored at −80 °C until further propagation. Serovar characterization of the clinical isolate was performed by sequencing the *omp1* gene as described previously [22]. *Chlamydia* strains were propagated and partially purified as described previously [23].

### 2.3. Preparation of AZT-Liposomes

AZT-liposomes were prepared by the modified proliposome method [19,24]. Briefly, the (phospho)lipids, AZT and Tween 80 (Table 1) were dissolved in ethanol (200 mg) during magnetic stirring (600 rpm, 50 °C). Then, 0.2 mL PB, pH 7.5 (pre-heated to the same temperature) was added, and the mixture was stirred to form an initial proliposome mixture. After cooling to room temperature, the AZT-proliposome mixture was transformed to AZT-liposomal dispersion by the drop-wise addition of PB, pH 7.5 to obtain the final volume of 10 mL. The dispersion was stirred (600 rpm) for 40 min at room temperature. All the liposomal dispersions were homogenized by ultrasonication for 10 s at 60 µW, using the Cole-Parmer Ultrasonic Homogenizer 4710 Series (Vernon Hills, IL, USA), and stored in the refrigerator at 4 °C. Prior to their use, the liposomes were incubated at room temperature and well mixed.

### 2.4. Size Measurements of AZT-Liposomes

The average diameters, polydispersity indexes (PDIs) and size distributions of the different AZT-liposomes were determined by dynamic light scattering, using a Horiba SZ-100 Nanoparticle Analyzer (Kyoto, Japan). Prior to the measurements, the liposomal dispersions were thermostated at room temperature and appropriately diluted with PBS, pH 7.4 to 0.02 mg/L (based on the AZT concentration in liposomes). All the measurements were carried out at 25 °C at a scattering angle of 90°, where the acquisition lag time of each measurement was 600. The results are expressed as the mean ± S.D (*n* = 5).

### 2.5. Zeta Potential Measurements of AZT-Liposomes

The zeta potentials of the different AZT-liposomes were determined on Zetasizer Ultra Red (Malvern Panalytical Ltd., Malvern, UK) using a folded capillary cell. The liposomal samples were previously diluted in PB, pH 7.5 to obtain a satisfactory quality factor (approximately to 0.02 mg/L). The measurements were performed at 25 °C. The results are expressed as the mean ± S.D (*n* = 5).

### 2.6. Liposomal Bilayer Elasticity Determination

The membrane elasticity of the different surface-charged liposomes was evaluated by previously reported method [25]. Briefly, the liposomal dispersions were continuously extruded (LiposoFast, Avestin, Ottawa, ON, Canada) at room temperature through a 100 nm pore-size membrane (*r_p_*) for 5 min by applying an external pressure of 2.5 bar, and the average diameter of the liposomes following extrusion (*r_v_*) as along with the mass of the extruded liposomes (*J*) were determined. For the assessment of AZT-liposomes, the non-encapsulated drug was pre-separated from the liposomally-encapsulated AZT by ultracentrifugation (2.7). A calculation of the degree of bilayer elasticity (*E*) was performed using the following equation:

$$E=J{\left(\frac{r_{v}}{r_{p}}\right)}^{2}$$

*r_v_* of the different liposomes was determined by dynamic light scattering on Zetasizer Ultra Red (Malvern Panalytical Ltd., Malvern, UK) at scattering angle of 90°. The liposomal dispersions were properly diluted by PB, pH 7.5 and the measurements were performed at 25 °C using disposable DTS0012 cuvettes. The results are expressed as the mean ± S.D (*n* = 3).

### 2.7. AZT Encapsulation Efficiency (EE) Determination

Encapsulation of AZT in the different surface-charged elastic liposomes was determined after separation of the non-encapsulated drug by ultracentrifugation. For this purpose, AZT-liposomes (1 mL) were diluted with 5 mL PB and ultracentrifuged (Beckman Optima LE-80 K Ultracentrifuge, Beckman Coulter Inc., Fullerton, CA, USA) for 1 h at 150,000× *g* (20 °C). The supernatant containing non-encapsulated AZT was separated from the pellet (liposomes). The pellet was washed with PB and ultracentrifuged under the same conditions. The amount of non-entrapped AZT was assessed in supernatants spectrophotometrically using a Varian Cary 50 UV/Vis spectrophotometer (Varian Australia Pty Ltd., Belrose, Australia) at 210 nm. The encapsulated AZT was calculated using the following equation:

$$EE\;\left(\%\right)=100-\left(\frac{non-encapsulated\;AZT}{total\;AZT}\right)\times 100$$

### 2.8. Cytotoxicity Measurement by 3-(4,5-Dimethyl-2-thiazolyl)-2,5-diphenyl-2H-tetrazolium Bromide (MTT) Assay

MTT assay was performed to find the maximum non-toxic concentration of the AZT-liposomes. HeLa 229 (ATCC) cells were placed into 96-well plates (Sarstedt) at a density of 4 × 10^4^ cells/well in 100 µL of minimal essential medium (MEM) with Earle salts supplemented with 10% heat-inactivated FBS (Gibco, Germany), 2 mmol/L L-glutamine, 1× MEM vitamins, 1× non-essential amino acids, 0.005% Na-pyruvate, 25 µg/mL gentamycin, 1 µg/mL Fungisone. The plates were incubated for 1 h at room temperature (RT) and then overnight at 37 °C, 5% CO_2_. The next day, when the cells reached a ~90% confluence, the medium was supplemented with serial 2-fold dilutions of the AZT-liposomes in three parallel wells for each concentration. Concentration ranges of 1–0.0156 µg/mL AZT for each type of AZT-liposomes, the corresponding concentrations of empty liposomes and free AZT were tested. The MTT (Sigma) labeling reagent (10 μL, final concentration 0.5 mg/mL) was added to each well, after 48 h incubation. The plate was incubated for 4 h at 37 °C, 5% CO_2_, then 100 µL of the solubilization solution (10% SDS in 1 N HCl) was added into each well and the plate was incubated overnight at 37 °C, 5% CO_2_. Labsystems Multiskan Ex 355 microtiter plate reader (Thermo Fisher Scientific, Waltham, MA, USA) was used to measure the optical density of the wells. The absorbance of the formazan product was measured at 540 nm. Three parallel measurements were performed.

### 2.9. Culture of HeLa 229 Cells and Direct qPCR Measurement of the Impact of AZT-Liposomes on C. trachomatis Growth

HeLa 229 cells (ATCC) were cultured in 96-well plates (Sarstedt, Nümbrecht, Germany) at a density of 4 × 10^4^ cells/well in 100 μL of minimal essential medium (MEM) with Earle salts supplemented with 10% heat-inactivated fetal bovine serum (FBS) (Gibco, Waltham, MA, USA), 2 mmol/L L-glutamine, 1× non-essential amino acids, 8 mmol/L HEPES, 25 μg/mL gentamycin and 1 µg/mL Fungisone. HeLa 229 cells were incubated overnight at 37 °C, 5% CO_2_ to get a 90% confluent cell layer. All the reagents were purchased from Sigma–Aldrich, unless otherwise indicated. HeLa cells were infected with *C. trachomatis* (multiplicity of infection (MOI) 1) for 1 h in 0.5% glucose containing medium without centrifugation. After infection, the cells were washed twice with PBS. The culture medium with cycloheximide was supplemented with the serial 2-fold dilutions of the AZT-liposomes and was added to triplicate wells. The cationic (+EL-AZT), anionic (−EL-AZT) and neutral (EL-AZT) AZT-liposomes as well as free AZT (6/4, *v*/*v*, ethanol/water solution) were diluted in culture medium with cycloheximide. Concentration ranges of 0.5–0.0002 μg/mL AZT for each AZT-liposomes and free AZT, with 2-fold dilutions were tested. The plates were incubated for 48 h at 37 °C, 5% CO_2_. Measurement of recoverable chlamydial growth was performed on McCoy (ATCC) cells. McCoy cells were transferred into wells of the 96-well plate at a density of 4 × 10^4^ cells/well in 100 µL of MEM, and were incubated overnight at 37 °C, 5% CO_2_ to obtain a 90% confluent cell layer. Before the infection, the wells were washed twice with 100 μL/well of PBS (pH 7.2). After the washing steps, 90 µL of culture medium with glucose was added to each well. For the determination of the recoverable inclusion forming units, 10 µL of the treated and *C. trachomatis* infected HeLa 229 cells were transferred onto the McCoy cells. The cells were centrifuged for 1 h at 800× *g* and were incubated for 48 h in cycloheximide-containing (1 µg/mL) growth medium. For direct qPCR, the supernatants of the infected HeLa 229 cells and McCoy cells were removed, and the cells were washed with 100 µL/well PBS twice. After the second wash, 100 μL Milli-Q (MQ) (Millipore, Billerica, MA, USA) water was added to each well and the samples were exposed to two freeze-thaw cycles with a quick freezing (−80 °C, 15 min) and a quick thawing on a plate shaker at room temperature. After the lysis, the cell lysates were thoroughly mixed and the mixed lysates were used as templates in the qPCR to measure the chlamydial genomic DNA. The qPCR was performed in a Bio-Rad CFX96 real time system (Bio-Rad, Hercules, CA, USA). The 5× HOT FIREPol^®^ EvaGreen^®^ qPCR Supermix (Solis BioDyne, Tartu, Estonia) master mix and *C. trachomatis* pykF gene-specific primer pairs were used for the amplification. Direct qPCR for chlamydial genome concentration measurements were performed as described previously [26]. Briefly, the composition of the reaction included 2 μL 5× HOT FIREPol^®^ EvaGreen^®^ qPCR Supermix, 1–1 μL forward and reverse primers (10 pmol each), 1 μL template and 5 µL MQ water. After a 10 min at 95 °C polymerase activation step, 40 PCR cycles of 20 s at 95 °C and 1 min at 64 °C were completed. Melting curve analysis was used to validate the specificity of amplification. For each sample the cycle threshold (Ct) value corresponding to the qPCR cycle where the amplification curve crossed the threshold was determined. Ct values were used to calculate the MIC and MBC values as described before [26].

### 2.10. Statistical Analysis

Statistical data analyses were completed using the GraphPad 9.2.0 Prism program (GraphPad Software, Inc., San Diego, CA, USA). For comparisons between two groups, Student’s *t*-test was used, while for comparisons of three or more groups one-way ANOVA and Tukey post-hoc test was used. Threshold for statistical significance was *p* < 0.05.

## 3. Results

### 3.1. Physico-Chemical Properties of the AZT-Liposomes

Neutral AZT-liposomes were prepared from egg lecithin containing at least 96% phosphatidylcholine (PC) with the addition of the edge activator, i.e., Tween 80 (neutral liposomes), while for the preparation of anionic or cationic liposomes negatively charged phospholipid (EPG) or cationic lipid (DODAB,) were additionally used (Table 1).

**Figure 1:**
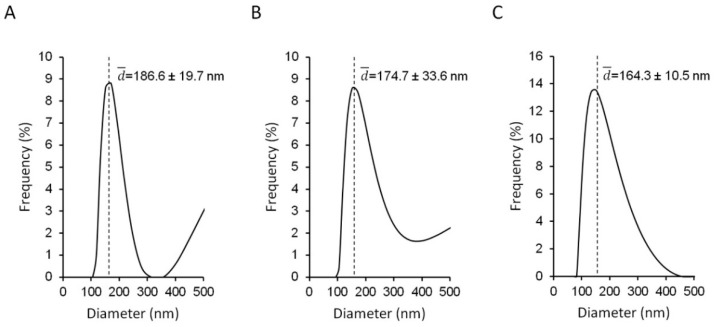
Size distributions and mean diameters of the different surface-charged AZT-liposomes (25 °C). (**A**), neutral (EL-AZT), (**B**), cationic (+EL-AZT) and (**C**), anionic (−EL-AZT) liposomes with azithromycin (AZT). The corresponding PDI values were: 0.48 ± 0.02 (**A**), 0.45 ± 0.04 (**B**) and 0.36 ± 0.05 (**C**), respectively. Data are mean ± S.D (*n* = 5).

Assessment of the physicochemical characteristics of the liposomes, such as the size, zeta potential and bilayer elasticity/rigidity, is of great importance as these properties influence the interactions of the liposomes with the microorganisms, as well as the cells and tissues in the biological environment, subsequently determining the therapeutic outcome. Elastic AZT-liposomes were prepared by a simple and reproducible method acceptable for scale up, followed by the short cycle of ultrasonication to homogenize the original AZT-liposomal dispersions. As a result, mean diameters of the originally prepared liposomes decreased and all the AZT-liposomes displayed moderate polydispersity (Figure 1A–C), characteristic for deformable (elastic) liposomes [27]. Those comprising the negatively charged EPG were the smallest (164 ± 10 nm). Liposomes incorporating positively charged DODAB were somewhat larger (175 ± 34 nm), but not significantly (ANOVA, *p* > 0.05), while liposomes, consisting of only neutral phospholipid, were the largest (187 ± 20 nm). The size distributions of AZT-liposomes (Figure 1) also indicate the presence of a smaller population of larger vesicles, typical for the selected preparation method [28], which is consistent with the previous studies [29,30] and could be the result of a short cycle of ultrasonication applied, too.

**Figure 2:**
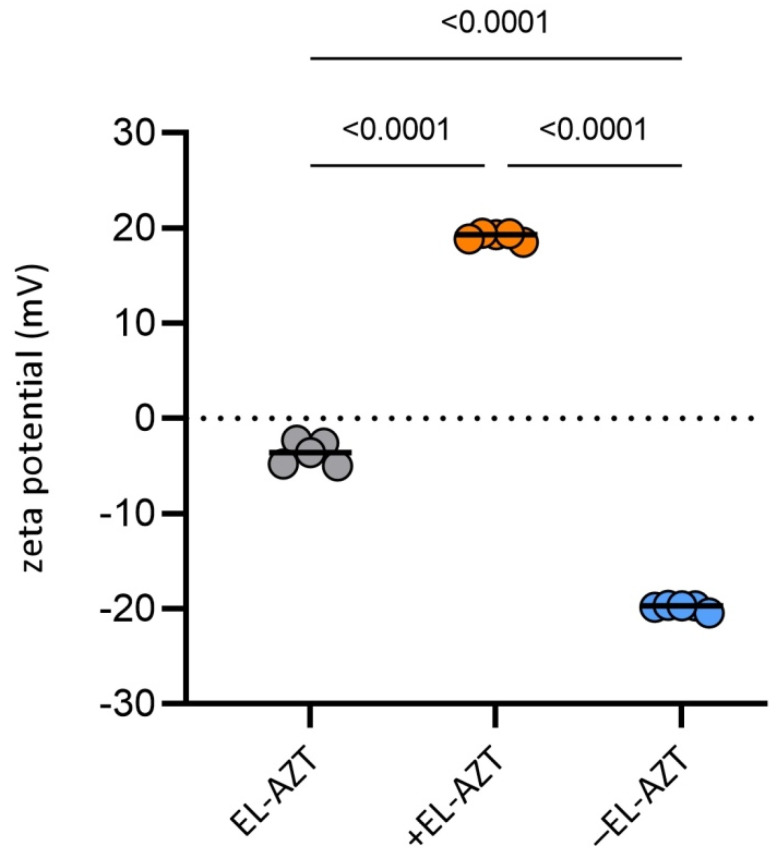
Zeta potentials of the different surface-charged AZT-liposomes. Data are mean and individual values (*n* = 5). Statistically significant differences with *p* values are shown. Statistical analysis was performed by one-way ANOVA with post-hoc Tukey test. EL-AZT, neutral liposomes with azithromycin; +EL-AZT, cationic liposomes with azithromycin; −EL-AZT, anionic liposomes with azithromycin.

Although phospholipid composition slightly affected the mean diameters of AZT-liposomes, it defined their surface charge (Figure 2). Embedding only 5% (*w*/*w*) EPG or DODAB in the liposomal bilayers significantly decreased or increased the zeta potentials of anionic or cationic AZT-liposomes (ANOVA, *p* < 0.05), respectively. These results are in accordance with the previous findings for deformable liposomes [25], and are considered beneficial regarding physical stability of the AZT-liposomes. Bilayers of all the liposomes consisted of 20% non-ionic single chain surfactant, i.e., polyoxyethylene (20) sorbitan monooleate (Tween 80), which has already been reported as the edge activator in deformable (elastic) liposomes [31]. The edge activator destabilizes the tight packing of phospholipids in the liposomal bilayers, making them deformable (flexible) and squeezable, thus subsequently enhancing permeation of the vesicles and/or encapsulated drug into/through the skin and cervicovaginal tissue [18,19,32].

**Figure 3:**
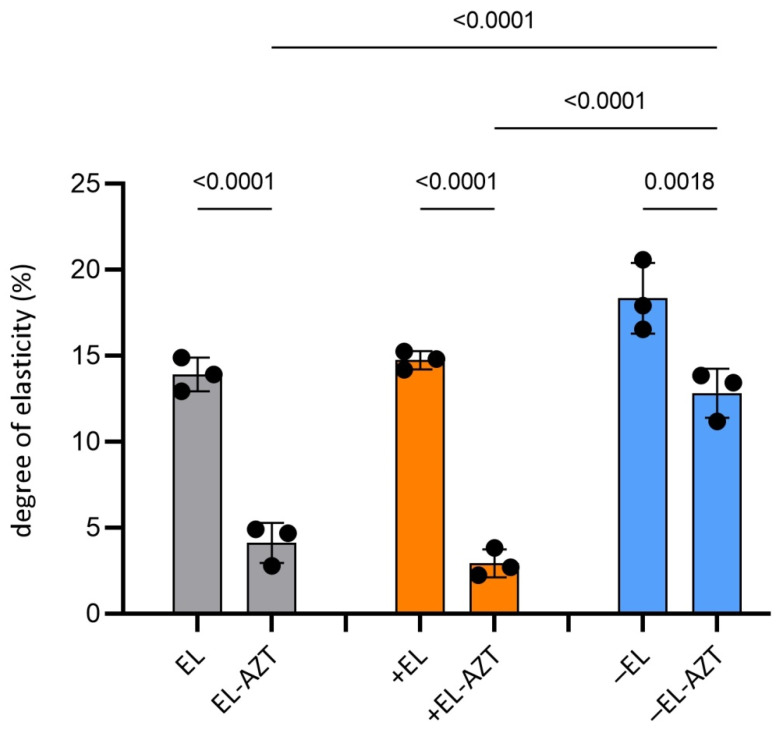
Degree of bilayer elasticity (E) of the different surface-charged liposomes. Data are mean ± S.D and individual values. (*n* = 3). Statistically significant differences between the corresponding empty liposomes and AZT-containing liposomes and between different AZT containing liposomes are shown. Statistical analysis was performed by one-way ANOVA with post-hoc Tukey test. EL, empty neutral liposomes; +EL, empty cationic liposomes; −EL, empty anionic liposomes; EL-AZT, neutral liposomes with azithromycin; +EL-AZT, cationic liposomes with azithromycin; −EL-AZT, anionic liposomes with azithromycin.

Evaluation of the liposomes’ deformability has proved that the liposomes prepared without AZT (empty liposomes) were highly elastic vesicles with a degree of bilayer elasticity (E) between 14 and 18 (Figure 3). However, the incorporation of AZT in cationic and neutral liposomes strengthened their membranes relative to the bilayers of the empty liposomes. This was confirmed by significantly decreased E values (3-4) (ANOVA, *p* < 0.05), while the membranes of anionic AZT-liposomes retained better their bilayer flexibility (E > 12).

**Figure 4:**
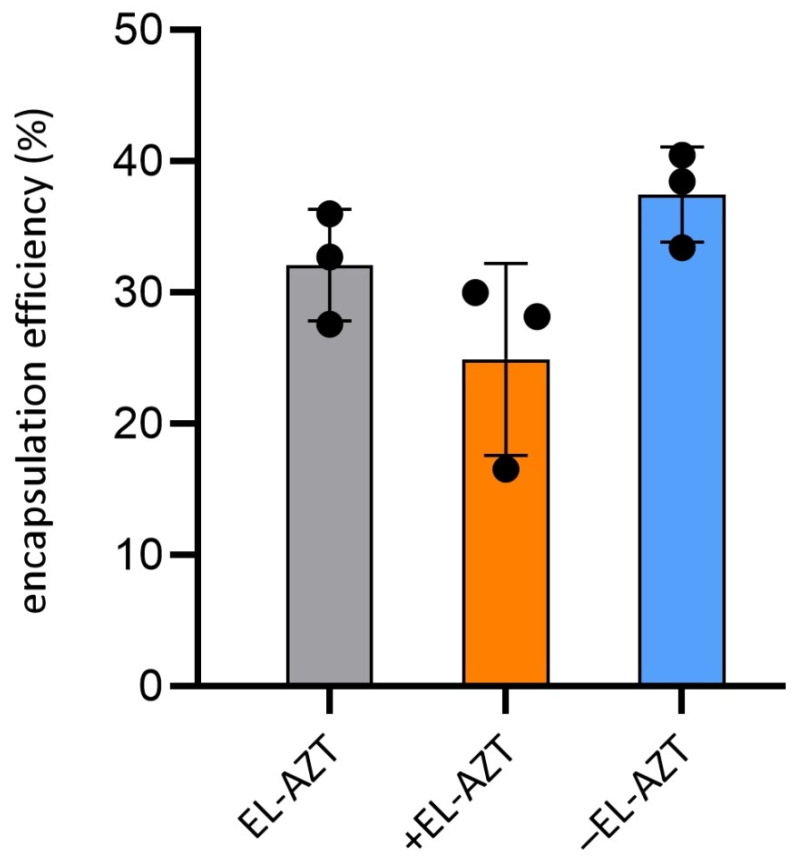
Encapsulation efficiency of AZT in the neutral (EL-AZT), cationic (+EL-AZT) and anionic (−EL-AZT) elastic AZT-liposomes. Data are mean ± S.D. and individual values (*n* = 3). Statistics analysis was performed by one-way ANOVA with post-hoc Tukey test. EL-AZT, neutral liposomes with azithromycin; +EL-AZT, cationic liposomes with azithromycin; −EL-AZT, anionic liposomes with azithromycin.

Separation of the non-encapsulated AZT was performed by ultracentrifugation method, which was previously reported to be similarly effective for separation AZT-liposomes as minicolumn centrifugation method [18]. Following the separation of the non-encapsulated AZT from the liposomally-encapsulated AZT, 25 and 37% of the drug was detected in cationic and anionic AZT-liposomes, respectively, while about 32% AZT was encapsulated in neutral liposomes (Figure 4). A tendency of higher AZT encapsulation in the anionic liposomes relative to the neutral and cationic AZT-liposomes is most likely a consequence of the interaction between the drug and the negatively charged EPG [33]. In comparison to the conventional liposomes, characterized by the rigid bilayers [25,27], the increased membrane deformability of the elastic liposomes could result in the leakage of the drug during storage. Therefore, to avoid these negative consequences, antichlamydial studies were performed with AZT-liposomes in which the non-encapsulated AZT was not removed from the liposomally-encapsulated drug. It is worth to note that the presence of the non-encapsulated AZT in AZT-liposomal dispersions contributes to the long-term stability of the nanoformulations by preventing leakage of the encapsulated drug from the vesicles. Drug leakage is considered a limitation; hence many commercial liposomal formulations are available as lyophilized products, which are converted to liposomal dispersions before use [11,34].

### 3.2. Cytotoxicity Measurement of the of AZT-Liposomes on HeLa 229 Cells

**Figure 5:**
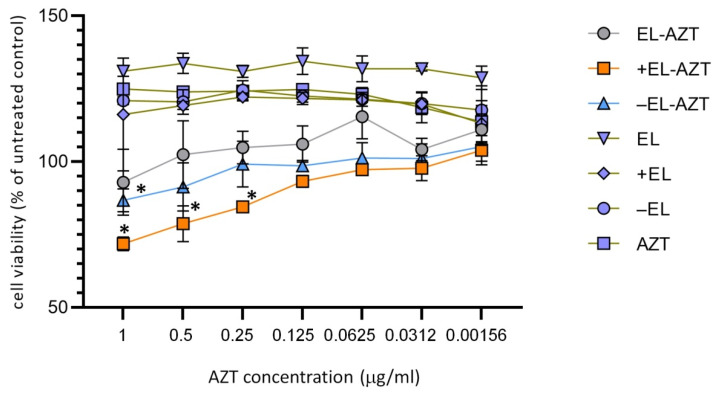
MTT assay of the HeLa 229 cells treated with the neutral, anionic and cationic AZT-liposomes. Viability of the liposomally-treated cells were compared to the untreated controls. Data are mean ± S.D. (*n* = 3). * Statistically significant MTT values of AZT-liposome treated cells compared to the untreated control cells (*t*-test, *p* < 0.05). EL-AZT, neutral liposomes with azithromycin; +EL-AZT, cationic liposomes with azithromycin; −EL-AZT, anionic liposomes with azithromycin; EL, neutral liposomes without azithromycin; +EL, cationic liposomes without azithromycin; −EL, anionic liposomes with azithromycin; AZT, free azithromycin.

**Figure 6:**
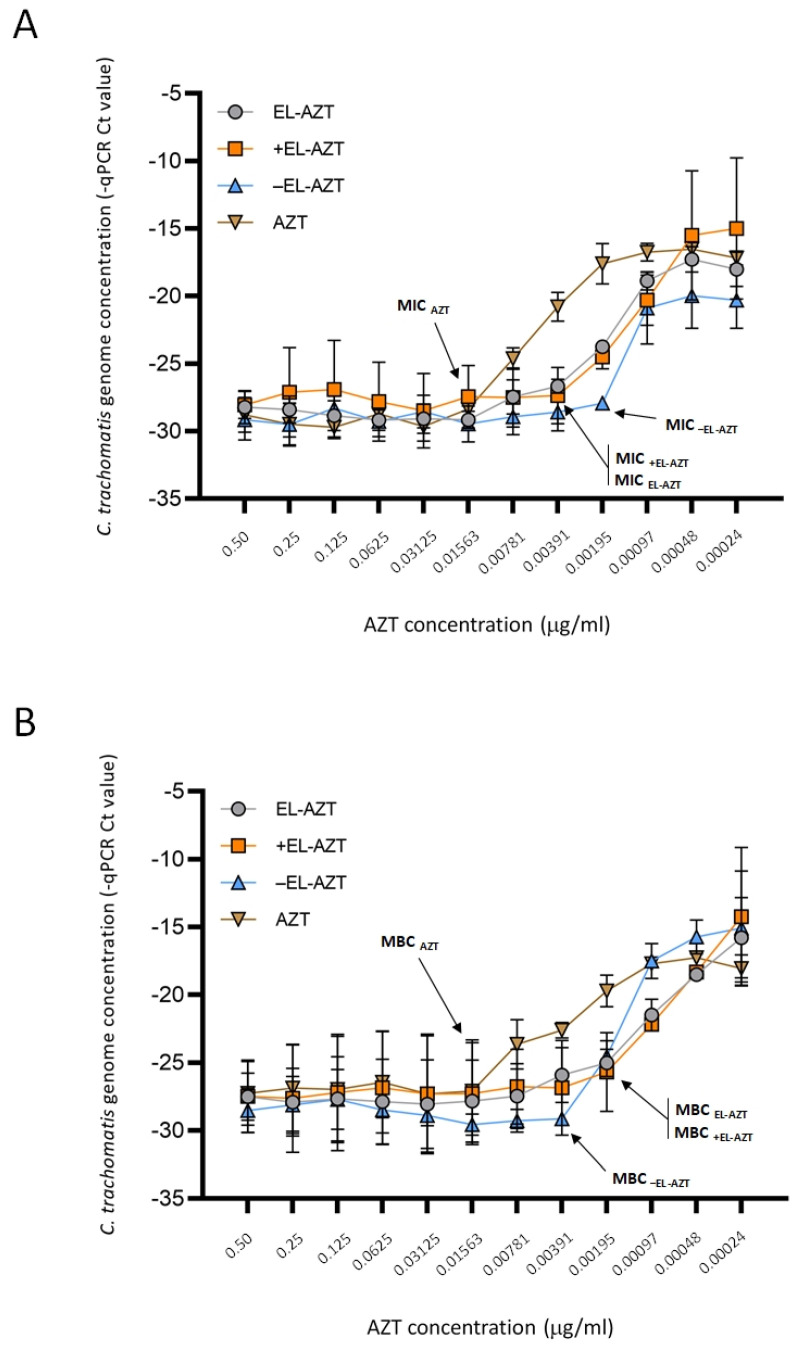
Impact of different types of AZT-liposomes on the growth of *C. trachomatis* serovar D. (**A**) Direct *C. trachomatis* growth in the presence of free AZT and liposomally-encapsulated AZT. (**B**) Recoverable *C. trachomatis* growth. Data are mean −qPCR Ct levels ± S.D. (*n* = 3). EL-AZT, neutral liposomes with azithromycin; +EL-AZT, cationic liposomes with azithromycin; −EL-AZT, anionic liposomes with azithromycin.

**Figure 7:**
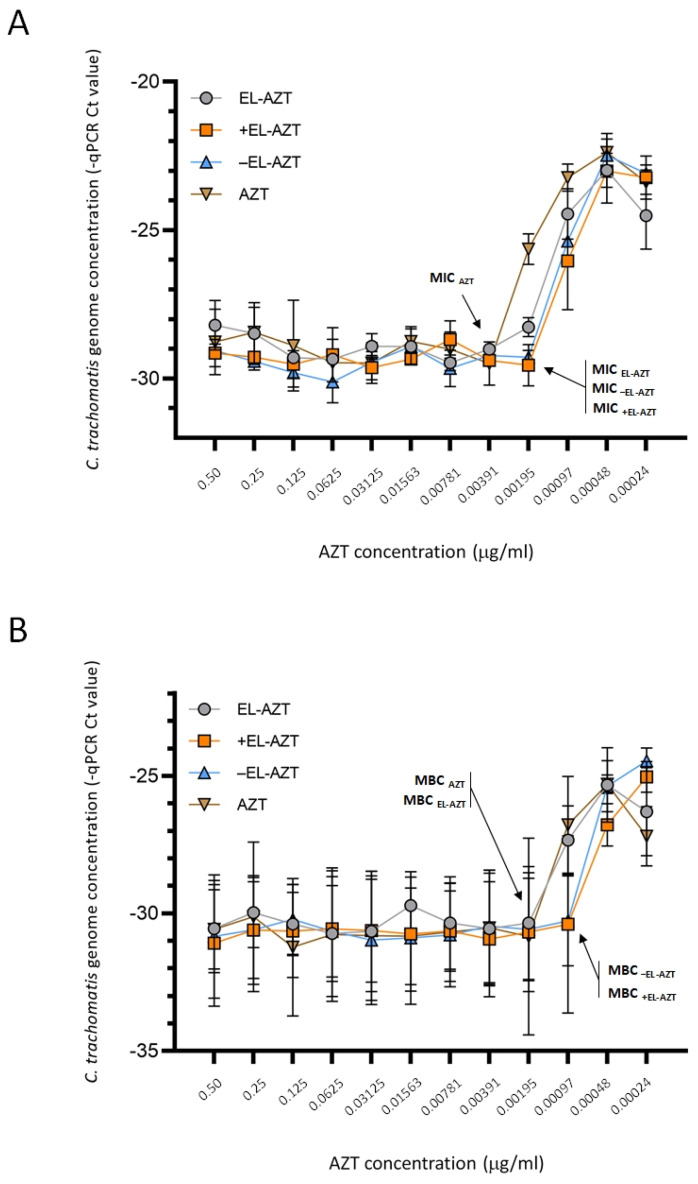
Impact of the AZT-liposomes on the growth of *C. trachomatis* serovar F clinical isolate. (**A**) Direct *C. trachomatis* growth in the presence of free AZT and encapsulated AZT. (**B**) Recoverable *C. trachomatis* growth. Data are mean −qPCR Ct levels ± S.D. (*n* = 3). EL-AZT, neutral liposomes with azithromycin; +EL-AZT, cationic liposomes with azithromycin; −EL-AZT, anionic liposomes with azithromycin.

*Chlamydiae* are obligate intracellular bacteria; hence the viability of the host cells may have an impact on the chlamydial growth. We performed an MTT assay to test whether the liposomes had a non-specific antichlamydial effect due to host cell cytotoxicity. MTT assay was performed with the AZT-liposomes at AZT concentrations ranging from 1 to 0.00156 μg/mL (Figure 5), the corresponding free liposomes and free AZT. Cell viability was assessed after 48 h of incubation. Cationic liposomes (+EL-AZT) had the highest impact on cell viability; the highest concentration without significant impact on cell viability was 0.125 μg/mL. It should be noted that this concentration was at least 32- up to 128-fold higher than the corresponding MIC and MBC values for cationic AZT-liposomes (Figure 6 and Figure 7), thus confirming their biocompatibility. The anionic (−EL-AZT) liposomes had a moderate impact on the cell viability with the highest non-toxic concentration of AZT 0.5 μg/mL, while the neutral (EL-AZT) liposomes had no significant impact on the cell viability at the tested concentrations. Empty liposomes and AZT alone did not decrease the cell viability in any of the tested concentrations.

### 3.3. Impact of AZT-Liposomes on C. trachomatis Growth

To evaluate the antimicrobial action of AZT-liposomes against *C. trachomatis* serovar, D/UW-3/CX laboratory strain and serovar F clinical isolate, infected HeLa 229 cells were treated with a 1:2 dilution series of AZT-liposomes (0.5–0.00024 μg/mL). Chlamydial genome content was measured 48 h post infection by direct qPCR and the MIC values were determined as described previously [26]. As shown in Figure 6A,B, all AZT-liposomes efficiently inhibited the growth of *C. trachomatis* serovar D. MIC values for anionic, cationic and neutral AZT-liposomes were lower compared to the 0.01563 μg/mL MIC for free AZT (Figure 6A). Among the tested liposomes, the most effective were the anionic liposomes with an 8-fold lower MIC than the MIC determined for free AZT. Neutral and cationic liposomes were equally effective in inhibiting the growth of *C. trachomatis* serovar D with a MIC value 4-fold lower in comparison to that obtained with free AZT (Figure 6A). The recoverable *Chlamydia* was also measured by centrifugation of the infected HeLa 229 cell lysates onto McCoy cells followed by the measurement of the chlamydial genome concentration 48 h post infection. The MBC value was calculated based on the recoverable chlamydial growth in McCoy cells in a similar manner to how the MIC was calculated. All the AZT-liposomes were effective in killing *C. trachomatis*; the MBCs for AZT-liposomes were lower compared to the 0.01563 μg/mL MBC of free AZT (Figure 6B). Neutral (EL-AZT) and cationic (+EL-AZT) liposomes exhibited the strongest activity against *C. trachomatis* serovar D with an MBC 8-fold lower than the MBC of the free AZT, while the anionic (−EL-AZT) liposomes had a 4-fold lower MBC relative to the free AZT (Figure 6B).

AZT-liposomes were also evaluated to determine their potential for inhibiting the growth and killing of the *C. trachomatis* serovar F clinical isolate (Figure 7A,B). Overall, the clinical isolate was more susceptible to AZT than the laboratory strain. Free AZT inhibited growth of serovar F at 0.00391 μg/mL (Figure 7A), in comparison to 0.01563 μg/mL determined for *C. trachomatis* serovar D. There were no differences in the MIC values between the different types of AZT-liposomes against serovar F clinical isolates. All the AZT-liposomes were equally effective with a MIC of 0.00195 μg/mL, which was 2-fold lower than the MIC determined for free AZT (Figure 7A). When AZT-liposomes were assessed for MBCs, those embedding only neutral phospholipid (EL-AZT) demonstrated the same 0.00195 μg/mL MBC value as free AZT, while cationic and anionic liposomes exhibited 2-fold lower MBC levels than the free AZT (Figure 7B). In vitro antichlamydial studies were performed up to 3 months after liposomal preparations. No microbiological contamination of the samples was observed in that period, nor did the storage affect the results. There were no significant differences between the results for the same type of the AZT-liposomes examined.

## 4. Discussion

In this study, we have tested several novel AZT-liposomal preparations to improve the efficacy of AZT against *C. trachomatis*. Our laboratory previously developed a direct qPCR method to quantitatively measure *C. trachomatis* growth as an alternative of the commonly used immunofluorescence detection and manual counting of chlamydial inclusions [26]. This method was used to measure the chlamydial genome content 48 h post infection in HeLa cells and the genome content of the recoverable *C. trachomatis* in McCoy cells. Hence, instead of direct infectious unit (IFU) and recoverable IFU, the two terms, direct growth and recoverable growth, were used. The measured MIC values refer to the inhibition of direct growth, while MBC values are characteristic of the inability of recovery—the inhibition of recoverable growth. Generally, our results showed that liposomal encapsulation enhanced the AZT activity by decreasing the MIC and MBC levels. MTT assays showed that the MIC (and MBC) concentrations were ~2 orders of magnitudes lower than the cytotoxic concentrations of the (charged) liposomes; therefore, the decrease of MIC levels were not due to a specific effect of the liposomes on the host cells. First, we measured the free AZT MIC and MBC for the laboratory strain *C. trachomatis* serovar D. The MIC value was 0.01563 μg/mL, which was similar to, or lower than the previously measured 0.03–0.5 μg/mL AZT values [35,36]. The MBC value was identical to the MIC for the laboratory strain in accordance with the previously measured similar AZT MIC and MBC values for *C. trachomatis* serovar D and other serovars [36,37]. Liposomal encapsulation of AZT decreased its MIC and MBC values even 4–8-fold. Interestingly, for neutral and cationic liposomes the MBC value was 2-fold lower than the MIC value. This was probably because during the MIC measurement the first sub-MIC concentration allowed a persistent state with ongoing chlamydial DNA synthesis, but these bacteria are not infectious and therefore the recoverable growth (represented by the MBC) at this concentration could not be detected. Indeed, Belland et al. have previously showed that the persistent, non-infectious form of *C. trachomatis* has intact DNA synthesis, but the bacterium cannot go through the normal division and re-differentiation to the infectious elementary body form [38]. As for the clinical serovar F strain, the free AZT MIC value was 2-fold lower than for the laboratory strain, while the MBC value was 2-fold lower than the MIC value, most likely due to the persistence inducing effect described above. The liposomal encapsulation had a moderate, but observable effect on the growth and recoverable growth of the clinical isolate, with a 2-fold lower MIC and MBC values. A notable exception was the neutral AZT-liposomes, which decreased the MIC value 2-fold, but could not change the MBC value compared to free AZT. Overall, these data show that there are differences between serovars and/or laboratory and clinical strains in the antibiotic susceptibility and in the efficacy of the liposomally-encapsulated antibiotic. Phenotypic differences between serotypes and clinical vs. laboratory strains have been shown before. A study by Thomas et al. on six different cell cultures demonstrated the propensity of the reference *C. trachomatis* type strain D/UW-3/CX to produce significantly higher amounts of infectious progeny in comparison to five clinical isolates from cervical swabs. Moreover, these clinical isolate used has also responded to AZT, penicillin and iron deprivation in a more pronounced manner than the reference strain revealing the potential for developing a persistence state more habitually in vivo [39].

Altogether, the surface charged liposomes were the most effective at improving the efficacy of AZT against both serovars. Although we expected cationic AZT-liposomes as the most effective due to opposite surface charge to the cell membrane, permitting extended contact on the cell surface, they were equally efficient as anionic AZT-liposomes. This result could be due to the lower content of DODAB (cationic lipid) in bilayers of cationic liposomes that do not provide strong interaction with the cell membrane, as well as lower encapsulation efficiency (25%) compared to the highest encapsulation achieved with anionic liposomes (37%). We assume that in this non-specific targeting approach, AZT-liposomes accumulate near the cells, serving as a depot and allowing higher local drug concentration. The released AZT passively diffuse across the cell membrane following concentration gradient and as a weak base accumulate inside the infected cells. A certain portion of the liposomes is likely to be internalized via endocytosis. The results of this study were different from our previous study, where the examined liposomes could not increase antichlamydial efficacy of AZT [19]. The difference in antichlamydial activity of the currently tested elastic AZT-liposomes compared to the deformable propylene glycol AZT-liposomes from our preceding study [19] is probably due to the presence of Tween 80, providing liposomal bilayer elasticity as well as increased permeation and internalization of AZT to the infected HeLa 229 cells. It has already been confirmed that deformable liposomes improved the delivery of the encapsulated drug deeper in the skin [27] and vaginal tissue [19]. The ratio of the edge activator inside the liposomal membrane as well as the type of the edge activator used influenced the membrane elasticity and permeation ability of elastic liposomes. By increasing the edge activator ratio, the membrane elasticity increases, and the release rate of the encapsulated drug can also be enhanced [40,41]. Therefore, by balancing the ratio of the edge activator and bilayer-building phospholipids, suitable liposomal nanoformulations could be obtained. Compared to the previous research [19], the edge activator in this study was present in a higher ratio (25%), contributing to enhanced elasticity. Another reason for the improved antichlamydial activity could be associated with the penetration enhancing effect of non-ionic surfactants, including Tween 80 [42,43], increasing the cellular delivery of AZT. However, further studies should be done to confirm these assumptions.

Our in vitro studies show the possibility that the elastic AZT-liposomes can be more effective for the treatment of *C. trachomatis* infections than the free AZT. They are biodegradable and biocompatible with the cervical cells, and significantly increase the antichlamydial activity of AZT. Additional studies such as drug release and storage stability measurements are needed to confirm the optimal liposomal preparation for the treatment of chlamydial infections.

## Figures and Tables

**Table 1:** Composition of AZT-liposomes.

Liposomes (Code)	EPC (mg)	EPG (mg)	DODAB (mg)	AZT (mg)	Tween 80 (mg)	PB, pH 7.5 (mL)
+EL-AZT	190	-	10	10	50	10
−EL-AZT	190	10	-	10	50	10
EL-AZT	200	-	-	10	50	10
+EL	190	-	10	-	50	10
−EL	190	10	-	-	50	10
EL	200	-	-	-	50	10

AZT, azithromycin dihydrate; DODAB, dimethyldioctadecylammonium bromide; EL, elastic liposomes composed of only neutral phospholipid (neutral liposomes); +EL, elastic liposomes embedding cationic lipid (cationic liposomes); −EL, elastic liposomes embedding anionic phospholipid (anionic liposomes); EPC, egg phosphatidylcholine; EPG, egg phosphatidylglycerol; PB, phosphate buffer; Tween 80, polyoxyethylene (20) sorbitan monooleate.

## Data Availability

The article contains all the data.
